# Doping im Breitensport

**DOI:** 10.1007/s00103-026-04245-3

**Published:** 2026-05-07

**Authors:** Pavel Dietz, Rolf Ulrich, Perikles Simon

**Affiliations:** 1 Institut für Arbeits‑, Sozial- und Umweltmedizin, Universitätsmedizin der Johannes Gutenberg-Universität Mainz, Obere Zahlbacher Straße 67, 55131 Mainz, Deutschland; 2 Fachbereich Psychologie, Kognition und Wahrnehmung, Eberhard Karls Universität Tübingen, Tübingen, Deutschland; 3 Abteilung Sportmedizin, Prävention & Rehabilitation, Institut für Sportwissenschaft, Johannes Gutenberg-Universität Mainz, Mainz, Deutschland

**Keywords:** Enhancement, Leistungssteigernde Substanzen, Prävalenz, Randomized-Response-Technik, Freizeitsport, Enhancement, Performance-enhancing substances, Prevalence, Randomized response technique, Recreational sports

## Abstract

Doping wird in der öffentlichen Wahrnehmung überwiegend dem professionellen Leistungssport und weniger dem Breitensport zugeschrieben. Dieser Diskussionsbeitrag behandelt Doping im Breitensport aus begrifflicher, methodischer und empirischer Perspektive. Etablierte Dopingdefinitionen, insbesondere die der Welt-Anti-Doping-Agentur, sind aufgrund ihres Verbands- und Wettkampfbezugs nur eingeschränkt auf breitensportliche Kontexte übertragbar. Für große Teile des Breitensports ist daher der Begriff des Konsums leistungssteigernder Substanzen angemessener.

Die Erfassung der Prävalenz dieses Konsums ist aufgrund des sensiblen Charakters und fehlender Kontrollmechanismen methodisch herausfordernd. Indirekte Befragungsverfahren reduzieren sozial erwünschtes Antwortverhalten und ermöglichen realistischere Schätzungen. Das Unrelated-Question-Modell (UQM) ist ein Randomized-Response-Verfahren, das durch vollständige Anonymisierung belastbare Prävalenzschätzungen sensibler Verhaltensweisen auf Populationsebene ermöglicht.

Empirische Befunde unter Verwendung des UQM bei Breitensporttreibenden aus Ausdauer‑, Fitness- und Bodybuilding-Sport zeigen, dass der Konsum leistungssteigernder Substanzen kein Randphänomen ist. Prävalenzschätzungen weisen auf eine relevante und über Jahre hinweg stabile Verbreitung hin. Enge Zusammenhänge bestehen zwischen nichttherapeutischem Schmerzmittelgebrauch und einer zunehmenden Medikalisierung der sportlichen Leistungsoptimierung. Hinweise auf Zugänge über medizinische Versorgungsstrukturen verdeutlichen die strukturelle Einbettung. Insgesamt stellt der Konsum leistungssteigernder Substanzen im Breitensport ein gesellschaftlich und gesundheitlich relevantes Handlungsfeld dar, das differenzierte Präventionsansätze sowie eine stärkere Berücksichtigung in der medizinischen Aus‑, Fort- und Weiterbildung erfordert.

## Abstract (en)

Doping is commonly perceived as being primarily associated with professional sports rather than recreational sports. This article is a discussion paper and addresses doping in recreational sports from conceptual, methodological, and empirical perspectives. Established doping definitions, particularly those of the World Anti-Doping Agency, are only partially transferable to recreational sports contexts due to their strong focus on organizational affiliation and competitive settings. For large segments of recreational sport, the concept of performance-enhancing substance use therefore provides a more appropriate description.

Assessing the prevalence of such use is methodologically challenging due to the sensitive nature of the topic and the lack of control mechanisms. Indirect survey techniques reduce socially desirable responding and yield more realistic estimates. The unrelated question model (UQM) is a randomized response technique that ensures full anonymity and enables robust prevalence estimates of sensitive behaviors at the population level.

Empirical findings using the UQM among recreational athletes in endurance, fitness, and bodybuilding contexts indicate that the use of performance-enhancing substances is not a marginal phenomenon. Prevalence estimates suggest a relevant and stable level over time. Strong associations are observed with non-therapeutic use of pain medication and an increasing medicalization of performance optimization. Evidence of access via healthcare structures highlights the structural embedding of this phenomenon. Overall, the use of performance-enhancing substances in recreational sport represents a socially and medically relevant field of action that requires differentiated prevention strategies and greater integration into medical education and training.

## Einleitung

Spätestens seit den prominenten Dopingaffären der 1990er- und 2000er-Jahre, beispielsweise um den ehemaligen Radprofi Lance Armstrong, das Team Telekom oder Berichte über systematisches Doping in bestimmten Verbänden und Ländern, ist das Thema Doping in den Medien, vor allem vor und während sportlicher Großevents, dauerhaft präsent. Diese mediale Aufmerksamkeit vermittelt jedoch häufig den Eindruck, Doping sei ein Phänomen, das nahezu ausschließlich im professionellen Leistungssport auftritt. Tatsächlich zeigen aktuelle Untersuchungen und gesellschaftliche Entwicklungen jedoch, dass der Gebrauch leistungssteigernder Substanzen und Methoden längst auch im Breitensport und anderen Bereichen der Gesellschaft angekommen ist [1–5].

Allerdings lässt sich das Thema Doping auf Grundlage der Erkenntnisse aus dem Leistungssport nicht einfach eins zu eins auf den Breitensport übertragen. Bereits die Definition von Doping, wie sie etwa von der Welt-Anti-Doping-Agentur (WADA) festgelegt wird, stößt im breitensportlichen Kontext an ihre Grenzen. Hinzu kommt, dass Breitensporttreibende häufig nur eingeschränkt über Doping, verbotene Substanzen und deren Risiken informiert sind. Anders als professionelle Athletinnen und Athleten, die in ein strukturiertes System aus medizinischer Betreuung, Aufklärung und Kontrollen eingebunden sind, verfügen viele Breitensporttreibende lediglich über fragmentarisches oder fehlerhaftes Wissen, das häufig von Medienberichten, Fitness-Mythen oder Empfehlungen aus dem sozialen Umfeld sowie aus digitalen Medien geprägt ist. Ein weiteres grundlegendes Problem besteht darin, die tatsächliche Prävalenz des Konsums leistungssteigernder Substanzen im Breitensport zuverlässig zu erfassen. Aufgrund fehlender Kontrollsysteme, sozial erwünschten Antwortverhaltens in empirischen Untersuchungen und heterogener sportlicher Milieus liegen oft nur grobe Schätzungen statt belastbarer Daten vor. Dadurch bleibt das Ausmaß des Problems unscharf und erschwert sowohl die wissenschaftliche Interpretation als auch die Entwicklung gezielter Präventionsmaßnahmen für den Breitensport.

Daher besteht das Ziel dieses Diskussionsbeitrags darin, das Phänomen Doping im Breitensport differenziert zu beleuchten. Dazu gehört erstens eine inhaltliche Auseinandersetzung mit der Definition von Doping im Kontext des Breitensports, die aufzeigt, inwiefern bestehende Begriffsbestimmungen nur eingeschränkt auf den Breitensport übertragbar sind. Zweitens erfolgt eine Darstellung indirekter Befragungsmethoden zur Erfassung der Prävalenz für den Konsum leistungssteigernder Substanzen im (Breiten‑)Sport, die den genannten Herausforderungen, insbesondere des sozial erwünschten Antwortverhaltens bei der Erforschung sensibler Themen, gerecht werden. Drittens wird eine Beschreibung der Prävalenz des Konsums leistungssteigernder Substanzen im Breitensport vorgenommen, um den aktuellen Forschungsstand zu systematisieren, das Ausmaß des Problems einzuordnen und die Rolle des Themas in Ausbildungskontexten zu diskutieren.

## Inhaltliche Auseinandersetzung mit der Definition von Doping im Kontext des Breitensports

Da in der Literatur keine einheitliche Definition des Begriffs „Breitensport“ zu finden ist, wird dieser häufig synonym mit „Freizeitsport“ verwendet und vor allem in Abgrenzung zum Leistungssport beschrieben. In sportwissenschaftlichen Texten erfolgt die Annäherung an den Begriff zumeist über charakteristische Merkmale wie offene Zugänglichkeit, geringe Leistungs- und Wettkampforientierung, eine auf Freizeit, Gesundheit und Wohlbefinden ausgerichtete Motivation sowie eine hohe Heterogenität der Teilnehmenden [6].

Gemäß dem aktuellen Code der Welt-Anti-Doping-Agentur (World Anti-Doping Agency, WADA) liegt Doping vor, wenn einer oder mehrere der in Artikel 2.1 bis 2.11 definierten Anti-Doping-Tatbestände erfüllt sind, darunter insbesondere der Gebrauch oder der Versuch des Gebrauchs verbotener Substanzen oder Methoden sowie weitere Verstöße wie das Umgehen von Kontrollen oder die Verweigerung der Probenabgabe [7]. Der Code gilt für Athletinnen und Athleten, die Mitglied eines internationalen Sportverbandes sind, der den Code anerkennt, die einem nationalen Sportverband angehören, der den Code anerkennt, oder die einem internationalen Verband zugehörig sind, der den Code anerkennt, die an Wettkämpfen oder Veranstaltungen teilnehmen, bei denen der Code als Regelwerk gilt, oder die einem Testpool, beispielsweise einer nationalen Anti-Doping-Agentur oder einem internationalen Verband, zugeordnet sind. Viele der hierin verankerten Regelungen betreffen Wettkampfbedingungen, Verbandszugehörigkeiten, Kontrollmechanismen oder Substanzlisten.

Für bestimmte Bereiche des Breitensports gelten eine oder mehrere dieser Bedingungen, für andere hingegen nicht, was die Anwendung des Dopingbegriffs im Breitensport erschwert. Hobbymäßige Läuferinnen und Läufer sowie Triathletinnen und Triathleten gelten als Breitensporttreibende. Nehmen diese an einem offiziell organisierten Wettkampf teil, beispielsweise einem Halbmarathon oder Triathlon, dessen Veranstalter den Code anerkennt oder gegebenenfalls Dopingkontrollen durchführt oder veranlasst, findet der WADA-Code Anwendung. In diesem Fall kann bei ihnen im Sinne der Dopingdefinition von Doping gesprochen werden. Für Breitensporttreibende, die ausschließlich in informellen Settings wie Fitnessstudios, Gesundheitskursen oder Freizeitgruppen aktiv sind, gilt der Code hingegen nicht, da zentrale Voraussetzungen für seine formale Anwendung, insbesondere Verbands- und Wettkampfbezug, hier nicht erfüllt sind.

Nicht alle Breitensporttreibenden fallen somit in die terminologisch und juristisch relevanten Kategorien, die den Gebrauch des Begriffs „Doping“ bestimmen. Dies hat entsprechende Konsequenzen für die kontextbezogene Verwendung des Dopingbegriffs: In diesem besonderen Setting ist er nicht eindeutig anwendbar. Daher verwenden wir im Zusammenhang mit dem Breitensport unter anderem in Anlehnung an Dresen et al. [8] nicht den Begriff des Dopings, sondern sprechen vom Konsum leistungssteigernder Substanzen und Methoden, die verschreibungspflichtig oder ausschließlich über den Schwarzmarkt verfügbar sind. Diese Begriffsverwendung basiert auf einer analytisch-empirischen Perspektive der Autoren und bildet einen wesentlichen, wenn auch nicht vollständigen Teil des Dopingspektrums in einer für Laien verständlichen Form ab.

## Indirekte Befragungsverfahren zur Erhebung der Prävalenz leistungssteigernden Substanzkonsums

### Sensible Forschungsfragen valide erforschen

Sensible bzw. sozial sensible Forschung befasst sich mit Fragestellungen, bei denen ein zustimmendes („Ja“) Antwortverhalten mit potenziell negativen Konsequenzen oder Nachteilen verbunden sein kann, entweder für die befragte Person selbst oder für die untersuchte Gruppe insgesamt [9, 10]. Dadurch besteht selbst bei anonym durchgeführten empirischen Untersuchungen die Gefahr, dass sensible Fragen nicht wahrheitsgemäß beantwortet werden und die tatsächliche Prävalenz entsprechender Verhaltensweisen oder Merkmale systematisch unterschätzt wird [11, 12]. Zu typischen sensiblen Themen zählen etwa häusliche Gewalt [13], sexuelle Neigungen und gesundheitsbezogene Aspekte [14, 15], politischer Aktivismus [16] oder, wie in diesem Beitrag im Fokus, Doping bzw. Substanzkonsum in Sport und anderen gesellschaftlichen Milieus [3, 5, 17–19]. Bereits 1965 postulierte Warner, dass Befragte bei Untersuchungen zu sensiblen Themen eher wahrheitsgemäße Angaben machen, wenn ihnen durch die Erhebungsmethodik absolute Anonymität garantiert wird, und entwickelte in diesem Zusammenhang erstmals die Randomized-Response-Technik (RRT; [20, 21]). Auf Grundlage Warners ursprünglichen Ansatzes entstanden in den folgenden Jahrzehnten zahlreiche Varianten der RRT. Zu den bekanntesten zählen unter anderem das Unrelated-Question-Modell [22], die Crosswise-Methode [23], die Item-Count-Technik [24], die Forced-Response-Methode [25, 26], das Cheater-Detection-Modell [27, 28] sowie der Stochastic Lie Detector [29].

Diese auf stochastischen Prinzipien beruhenden indirekten Befragungstechniken gewährleisten den Teilnehmenden vollständige Anonymität. Das bedeutet: Bei Verwendung einer RRT lässt sich das individuelle Antwortverhalten zu keinem Zeitpunkt rekonstruieren. Es lässt sich lediglich, bei ausreichender statistischer Power [30], ein Schätzwert für die Prävalenz des sensiblen Merkmals in der Gesamtstichprobe bestimmen. Eine Metaanalyse von 38 Validierungsstudien zeigt, dass RRTs validere Ergebnisse bei der Untersuchung sensibler Fragestellungen liefern als direkte Befragungsmethoden, etwa klassische anonyme Surveys oder Interviews [31]. Zur vertiefenden Auseinandersetzung mit der Validität von RRTs sei darüber hinaus die Arbeit von Moshagen et al. empfohlen [32].

### Das Unrelated-Question-Modell

Beim Unrelated-Question-Modell (UQM) werden die Befragten mithilfe eines „Randomisierers“ (z. B. einer Münze, eines Würfels oder eines Kartenspiels) zufällig einer von 2 wahrheitsgemäß zu beantwortenden Fragen zugewiesen: entweder einer neutralen, nichtsensiblen Frage (A) oder einer sensiblen Frage, die das interessierende Merkmal enthält (B, z. B. eine Frage zum Doping). Die Zuweisung erfolgt mit festgelegten Wahrscheinlichkeiten, sodass ein Teil der Befragten die neutrale und ein größerer die sensible Frage beantwortet.

Im Rahmen früherer Studien unserer Arbeitsgruppe [2–4] wurde diese Randomisierung auf ein Paper-and-pencil-Verfahren übertragen. Die Befragten wurden gebeten, bei der Randomisierung an einen beliebigen Geburtstag zu denken. Lag dieser gedachte Geburtstag in den ersten 10 Tagen eines Monats, sollten sie Frage (A) beantworten, andernfalls Frage (B). Auf diese Weise wurde eine klar definierte, für die Forschenden jedoch nicht nachvollziehbare Zufallszuweisung realisiert, deren Wahrscheinlichkeit berechnet werden kann. Die neutrale Frage war wiederum so gewählt, dass sie mit einer bekannten Wahrscheinlichkeit mit „Ja“ oder „Nein“ beantwortet wird. In den genannten Studien lautete sie beispielsweise: „Liegt der Geburtstag, an den Sie eben gedacht haben, in der ersten Jahreshälfte, also vor dem 1. Juli?“ Für beide Fragen standen ausschließlich die Antwortoptionen „Ja“ oder „Nein“ zur Verfügung. Da die Forschenden nicht wissen können, an welchen Geburtstag die Teilnehmenden gedacht haben, ist nicht erkennbar, ob sich eine gegebene Antwort auf die neutrale oder die sensible Frage bezieht. Die Befragung ist somit für den einzelnen Teilnehmenden vollständig anonym, da die Randomisierung ausschließlich im Kopf der befragten Person erfolgt.

Auf Grundlage der bekannten Zuordnungswahrscheinlichkeiten und der Verteilung der Antworten in der Stichprobe kann anschließend die Prävalenz des sensiblen Merkmals auf Populationsebene geschätzt werden, ohne Rückschlüsse auf einzelne Personen zuzulassen. Ein konkretes Rechenbeispiel sowie eine ausführliche Darstellung der Befragungsmethodik können unter anderem bei Franke et al. sowie Dietz et al. nachvollzogen werden [2, 4, 17].

Ein Nachteil dieses Modells ist jedoch, dass es in der Originalfassung nicht möglich ist, Schummler zu identifizieren, also Personen, die die Anweisungen der Befragung missachten und stattdessen selbstschützende Antworten geben. Reiber et al. haben daher eine verfeinerte Version des UQMs entwickelt, die darauf ausgelegt ist, den Anteil geschummelter Antworten zu erfassen [33]. Diese Version konnte beispielsweise erfolgreich zur Schätzung der Prävalenzen häuslicher Gewalt angewandt werden [34].

## Prävalenz für den Konsum leistungssteigernder Substanzen im Breitensport

Die nachfolgend dargestellten Prävalenzbefunde beziehen sich auf spezifische sportliche Settings (Ausdauersport, Fitnesssport) und Substanztypen und sind daher als exemplarische Einblicke in spezifische Teilbereiche des Breitensports zu verstehen und decken weder den Dopingbegriff noch den Breitensport in Gänze ab. Im Ausdauersport zeigt sich seit Jahren, dass der Konsum leistungssteigernder Substanzen keineswegs auf den Spitzensport beschränkt ist. In diesem Kontext greifen Athletinnen und Athleten in relevantem Umfang zu Substanzen und Methoden, um ihre körperliche Leistungsfähigkeit gezielt zu beeinflussen. Eine der bislang umfangreichsten Untersuchungen unter 2997 weitestgehend Breitensporttreibenden im Triathlon schätzte mittels RRT eine 12-Monats-Prävalenz von 13,0 % für den Konsum körperlich leistungssteigernder Substanzen wie anabolen Steroiden, Erythropoetin (EPO) oder Stimulanzien [3]. Diese Ergebnisse werden durch weitere Studien bestätigt und erweitert. Eine weitere groß angelegte Erhebung unter 1953 deutschen Triathletinnen und Triathleten, die alle gängigen Wettkampfdistanzen einschloss, identifizierte eine Jahresprävalenz von 7,0 % für den Konsum körperlich leistungssteigernder Substanzen. In dieser Studie waren Merkmale wie höheres Alter, langjährige Ausdauersporterfahrung oder größere Trainingsumfänge mit einem erhöhten Risiko für den Konsum leistungssteigernder Substanzen assoziiert [35]. Eine weitere, bis dato unveröffentlichte, aber in den Medien und sozialen Netzwerken hinreichend thematisierte empirische Untersuchung unter Verwendung von RRT beim Challenge Roth 2024, einem der weltweit größten Triathlon-Events, ergab eine Jahresprävalenz von 11 % für den Konsum leistungssteigernder Substanzen. Dieses Ergebnis liegt zwischen den in früheren Studien eruierten Werten und deutet darauf hin, dass der Substanzgebrauch unter Breitensporttreibenden im Ausdauersport weiterhin ein relevantes und konstantes Phänomen ist.

Die wissenschaftlichen Befunde werden zusätzlich durch journalistische Recherchen untermauert. So zeigt die investigative Recherche des Bayerischen Rundfunks im Rahmen des Challenge Roth 2024 mit dem Titel „So leichtfertig dopen Amateursportler“, dass im Breitensport nicht nur ein beträchtlicher Anteil der Sporttreibenden zu leistungssteigernden Substanzen greift, sondern dass einzelne Personen diese sogar über offizielle ärztliche Verschreibungen erhalten. Die Recherche schildert einen Fall, in dem ein Amateursportler Erythropoetin (EPO) nicht über den Schwarzmarkt, sondern über eine reguläre ärztliche Verordnung bezog, ohne dass eine medizinische Indikation vorlag. Auch eine wissenschaftliche Analyse zu den Gruppen, Motiven und Bezugsquellen von Dopingsubstanzen im Fitnesssport hatte unter Ermittlung einer realistischen Lebenszeitprävalenz von 14 % ergeben, dass das Gesundheitssystem einen wichtigen Beitrag zur Versorgung mit Dopingsubstanzen leistet [36]. Allerdings wurde die Erhebung vor über 2 Jahrzehnten im Fitnessbereich noch mittels einfacher Fragebögen ohne RRT durchgeführt. Seit den gesetzlichen Verschärfungen und der Einführung des Straftatbestandes „Doping“ steht zu befürchten, dass diese Sachverhalte aufgrund der stärkeren Stigmatisierung nicht mehr so einfach wissenschaftlich untersucht werden können. Die Unterstützung durch das Gesundheitssystem wirft ein Schlaglicht auf strukturelle Faktoren, die den Zugang zu leistungssteigernden Substanzen erleichtern und die Verbreitung im Breitensport zusätzlich begünstigen.

Parallel dazu stellt sich der nichttherapeutische Gebrauch von Schmerzmitteln als eigenständiges Problemfeld dar, zumal die Einnahme von Schmerzmitteln wie Ibuprofen, Diclofenac, ASS, Naproxen oder Paracetamol sowohl im Training als auch im Wettkampf erlaubt ist und diese nicht auf der WADA-Verbotsliste stehen. Eine Befragung von 3913 Marathonteilnehmenden zeigte, dass über die Hälfte der Teilnehmenden vor dem Wettkampf Schmerzmittel einnahm, häufig ohne medizinische Beratung. Der Schmerzmittelgebrauch erwies sich als gesundheitlich riskant, da er mit hohen Raten gastrointestinaler Beschwerden, erhöhter Blutungsneigung und kardiovaskulären Problemen einherging, während zugleich keine objektiven Leistungs- oder Erholungsgewinne nachweisbar waren [37, 38]. Die enge Verbindung zwischen Schmerzmittelgebrauch und dem Konsum leistungssteigernder Substanzen, wie sie auch in der Studie von Seifarth et al. [35] oder Dietz et al. [39] beobachtet wurde, verweist darauf, dass im Breitensport eine breite Medikalisierung stattfindet.

Auch im Fitness- und Bodybuilding-Bereich ist der Konsum leistungssteigernder Substanzen keine Seltenheit. Bereits frühe Arbeiten der 1980er- und 1990er-Jahre belegten eine hohe Nutzung insbesondere anaboler Steroide, obwohl deren Risiken bekannt sind [40–43]. Jüngere Studien mittels RRT zeigten Prävalenzen zwischen 12 % und 21 % unter Fitnessstudiobesuchern für leistungssteigernde Substanzen sowie über 40 % für den Konsum illegaler Drogen [36, 44–46]. Besonders auffällig war, dass ein erheblicher Teil der Fitnesssporttreibenden in diesen Studien mehrere Substanzen kombinierte und ein beachtlicher Anteil die Präparate über medizinische Zugangswege bezog. Im Bodybuilding zeigt sich darüber hinaus eine ausgeprägte kulturelle Differenzierung. Das „Natural Bodybuilding“ grenzt sich explizit vom dopingunterstützten Training ab und betont die körperliche Leistungsentwicklung ohne pharmakologische Hilfsmittel. Demgegenüber wird im nichtnaturalen (nichtdopingfreien) Bereich der Konsum leistungssteigernder Substanzen zunehmend offen kommuniziert und als legitimer Bestandteil spezifischer Körper- und Leistungsziele dargestellt. Die Sichtbarkeit dieser Praktiken, insbesondere in sozialen Medien, trägt zur Normalisierung des Substanzgebrauchs bei und kann die Einbettung in trainingstechnische Selbstoptimierungsstrategien verstärken.

Insgesamt verdeutlichen die vorliegenden Studien und Recherchen, dass der Konsum leistungssteigernder Substanzen im Breitensport ein strukturell verankertes Phänomen zu sein scheint, das durch individuelle Leistungsorientierung, mediale Einflussfaktoren und subkulturelle Normen geprägt wird. Die Prävalenzdaten aus Triathlon, Fitness und Bodybuilding zeigen, dass die Nutzung nicht nur konstant geblieben ist, sondern sich in Teilen der sportlichen Szene sozial normalisiert hat. Damit wird deutlich, dass der Konsum leistungssteigernder Substanzen ein eigenständiges und gesundheitlich relevantes Handlungsfeld darstellt, das dringend differenzierte Präventions- und Aufklärungsstrategien erfordert.

## Fazit

Der Konsum leistungssteigernder Substanzen ist im Breitensport verbreitet. Allerdings ist anzumerken, dass sich die in diesem Beitrag berichteten Studien vor allem auf wettkampfbezogene Kontexte im Ausdauersport und im Fitnesssport beziehen. Der Beitrag bildet den Breitensport insgesamt nicht vollständig ab. Gleichwohl ist davon auszugehen, dass dem Konsum leistungssteigender Substanzen im Vergleich zur deutlich höheren Relevanz des Alkoholkonsums für Selbst- und Fremdgefährdung eine eher nachgeordnete Bedeutung zukommt [47]. Das Besondere am Konsum leistungssteigernder Substanzen ist seine gesellschaftliche Verankerung. Die im Beitrag dargestellten Befunde aus dem Ausdauer‑, Fitness- und Bodybuilding-Sport sowie die Zusammenhänge mit dem nichttherapeutischen Gebrauch von Schmerzmitteln zeigen, dass leistungsbezogener Substanzkonsum im Breitensport kein isoliertes Randphänomen ist. Ältere Befunde weisen zudem darauf hin, dass auch medizinisches Personal eine Rolle spielen kann, wenn ein solcher Konsum in der Versorgung nicht erkannt, nicht thematisiert und nicht wirksam verhindert, sondern durch unkritische Verschreibungen begünstigt wird. Vor diesem Hintergrund sollte das Thema in medizinischen Aus‑, Fort- und Weiterbildungsformaten aufgegriffen werden, um Prävention, Beratung, Früherkennung sowie eine verantwortungsvolle Verordnungspraxis zu verbessern.

## Data Availability

Bei dem Artikel handelt es sich nicht um eine Originalarbeit und es liegen keine Originaldaten zugrunde.
